# Porphyrin-nitrogen carbon dot composites for high-performance organic light-emitting diodes

**DOI:** 10.1038/s41598-026-35190-5

**Published:** 2026-01-29

**Authors:** Zoi Georgiopoulou, Maria Eleni Rizou, Apostolis Verykios, Anastasia Soultati, Georgios Chatzigiannakis, Theodoros M. Triantis, Alexander Chroneos, Kalliopi Ladomenou, Athanassios G. Coutsolelos, Maria Vasilopoulou

**Affiliations:** 1 Institute of Nanoscience and Nanotechnology, National Center for Scientific Research ‘Demokritos’, Agia Paraskevi, 15310 Athens, Greece; 2 Solid State Physics Section, Department of Physics, National and Kapodistrian University of Athens, 15784 Panepistimioupolis, Zografos, Athens, Greece; 3 Department of Materials, Imperial College, London, SW7 2AZ UK; 4 Department of Electrical and Computer Engineering, University of Thessaly, 38221 Volos, Greece; 5 Hephaestus Laboratory, School of Chemistry, Faculty of Sciences, Democritus University of Thrace, 65404 Kavala, Greece; 6 Laboratory of Bioinorganic Chemistry, Department of Chemistry, University of Crete, Voutes Campus, 70013 Heraklion, Crete, Greece; 7 Foundation for Research and Technology (FORTH), Institute of Electronic Structure and Laser (IESL), 70013 Heraklion, Greece

**Keywords:** Porphyrin, Carbon dots, OLEDs, Electron transport layer, Materials science, Nanoparticles

## Abstract

Fluorescent organic light-emitting diodes (OLEDs) still face major obstacles to combining high efficiency with solution processability and energy-sustainable operation. In this work, a tetra-carboxyphenyl porphyrin–nitrogen carbon dots (TCPP-NCDots) derivative is introduced as a solution-processable electron transport layer (ETL) in OLEDs based on a green–yellow emissive polymer. The structural, optical, and electronic properties of the TCPP-NCDots ETL are comprehensively characterised by ultraviolet–visible absorption spectroscopy, steady-state photoluminescence, Fourier-transform infrared spectroscopy, atomic force microscopy, and cyclic voltammetry. These studies reveal a synergistic effect of porphyrin functionalisation on the carbon dots, which improves electron injection and transport toward the emissive layer. At an optimum concentration of 1 mg mL^−1^, the TCPP-NCDots ETL yields a 21.6% enhancement in external quantum efficiency compared with the reference device without an ETL, while simultaneously suppressing efficiency roll-off. The optimised devices also maintain stable operation under ambient conditions, highlighting TCPP-NCDots as a promising, sustainable ETL platform for next-generation fluorescent OLEDs.

**Supplementary Information:**

The online version contains supplementary material available at 10.1038/s41598-026-35190-5.

## Introduction

Organic Light-Emitting Diodes (OLEDs) have become central to modern optoelectronics, offering high contrast, precise color rendering and self-emissive operation^1,2^. These characteristics enable the development of thin, flexible, and energy-efficient devices that are now widely used in advanced display and lighting technologies^3,4^. Current research efforts focus on enhancing device efficiency, stability and large-scale manufacturability by introducing new functional materials and optimizing charge transport layers^5–7^. Within this framework, green fluorescent OLEDs have gained renewed attention^8^. Their simple device architecture eliminates the need for costly heavy metals employed in phosphorescent systems, while their compatibility with solution processing enables low-cost and environmentally sustainable fabrication^9–11^. Recent studies have demonstrated F8BT(Poly(9,9-dioctylfluorene-alt-benzothiadiazole))-based green fluorescent OLEDs in flexible slot-die coated structures^12–14^, microcavity architectures^15,16^, hybrid organic–inorganic systems^17^, optical and environmental sensors^15^, circularly polarized OLEDs^18,19^, and integrated photonic circuits ^20,21^, underscoring their versatility and technological relevance.

Achieving high performance in OLEDs depends on precise control of charge injection and transport. The electron transport layer (ETL) plays a decisive role by enabling efficient electron injection, supporting balanced carrier transport and suppressing exciton quenching at critical interfaces, which lowers turn-on voltages and improves luminance and lifetime^22–24^. A wide range of ETL materials has been developed to meet these requirements. Metal oxides such as ZnO and TiO_2_ are attractive due to their high transparency, compatibility with solution processing, and suitability for flexible, large-area fabrication^25–27^. Small molecules including Alq_3_, BPhen, TPBi, BCP, PBD, TAZ, B3PYMPM, and PO-T2T have long served as efficient electron-transport and exciton-blocking layers in high-performance OLEDs^28–30^. Although traditionally vacuum-deposited, TPBi and related materials can also be processed from solution using orthogonal solvents, enabling integration into polymeric and hybrid architectures^31,32^. Conjugated polymers provide fully solution-processed alternatives, combining good electron mobility with straightforward film formation^33,34^. Interlayers like PEIE, Cs_2_CO_3_, CsF, and Liq are often used to tune electrode work functions and lower injection barriers, improving charge balance and overall performance^35–37^. In green fluorescent OLEDs, where only singlet excitons contribute to emission, ETL engineering is particularly critical. These developments highlight the central role of ETL selection in determining device performance and motivate the search for new solution-processable alternatives, including porphyrins and carbon-based nanostructures.

Porphyrins are nature-derived complexes with carbon-rich conjugated systems. They have the potential to be incorporated into OLED devices, offering excellent charge transport and strong light-matter interactions. This is attributed to their unique macrocyclic π-conjugated system and a structure based on a tetrapyrrolic ring^38,39^. Their extended π-electron delocalization facilitates charge mobility and enables efficient transport of both electrons and holes^40,41^. Their planar, rigid backbone enhances orbital overlap and prevents structural distortions^42^. Additionally, the peripheral functionalization of this molecular system allows control over their electronic states^43^. The ability to modulate their HOMO–LUMO energy levels enables optimized charge injection and transport in optoelectronic applications^44,45^. Their delocalized electronic structure also supports efficient exciton generation and migration, which is crucial for high-performance OLED devices. Initially, porphyrins were investigated as phosphorescent sensitizers or dopants in the emissive layer, aiming to enhance triplet exciton harvesting and improve device quantum efficiency^46,47^. Subsequent studies revealed a different functional role for porphyrins when incorporated at the interface between the cathode and the emissive layer. In these configurations, porphyrins improved electron injection and reduced interfacial energy barriers, exhibiting behavior analogous to electron-transport layers^48–51^.

In parallel, carbon dots (CDots) have emerged as a prospective class of nanomaterial for OLED applications^52^. They are derived from cost-effective and sustainable synthesis methods, mostly from organic materials^53^. Their low toxicity and heavy metal-free composition position them as an efficient alternative to traditional quantum dots such as CdSe or perovskite QDs^54^. The sp^2^-hybridized carbon structure of CDots facilitates charge transport^55^. Their nanoscale size and tunable surface chemistry enable precise control of the electronic states and the light emission^56^. The versatile integration of CDots into OLED architectures, whether as light-emitting or interfacial layers, has also been demonstrated by Wang et al.^57^. This work showed that CDots function as both electron donors and acceptors, enhancing charge transport and minimizing energy losses in optoelectronic devices. Additionally, Zhao and Tan, in their review, investigated the application of CDots as an emissive layer (EML), successfully tuning photoluminescence, optimizing charge transport and achieving good stability^58^. Furthermore, Alam et al. explored the use of CDots derived from banana leaves as ETL in OLEDs and found that CDots reduce turn-on voltage, enhance charge injection and improve device stability by increasing electron mobility^59^.

In a previous study^60^, nitrogen-functionalized carbon dots (NCDots) were prepared from low-cost organic precursors using a bottom-up approach^61^. The structure included sodium carboxylate and amino-terminated carboxylic acid groups. When used as electron transport layers (ETLs) in OLEDs, NCDots led to a threefold increase in both luminous and external quantum efficiency. In this study, we advance this approach by introducing a novel electron transport layer (ETL) for fluorescent OLEDs, based on tetra-carboxyphenyl porphyrin-functionalized nitrogen-doped carbon dots (TCPP-NCDots). This study focuses on the combination and synergistic interaction between porphyrin and NCDot molecules, aiming to further enhance electron injection and transport in solution-processed devices. In this context, Achilleos et al. confirmed the formation of a stable nano-sized hybrid upon the covalent connection of free-base TCPP with NCDots^62^. The material retains the characteristic porphyrin absorption bands and the excitation-dependent emission of NCDots. It remains photostable under continuous irradiation. Its metal-free nature, combined with these optical and structural characteristics and low-cost synthesis, makes it a promising candidate for ETL applications. Through a comprehensive investigation using UV–Vis absorption, photoluminescence (PL), Fourier-transform infrared (FTIR) spectroscopy, atomic force microscopy (AFM) and cyclic voltammetry (CV), we elucidate the structural, electronic, and optical properties of TCPP-NCDots. OLED devices with TCPP-NCDots in the ETL show significant improvements over the reference. This highlights the role of TCPP porphyrins in enhancing efficiency and opens new paths for material-driven OLED design.

## Results

### Influence of TCPP-NCDots on the surface of F8BT

**Fig. 1:**
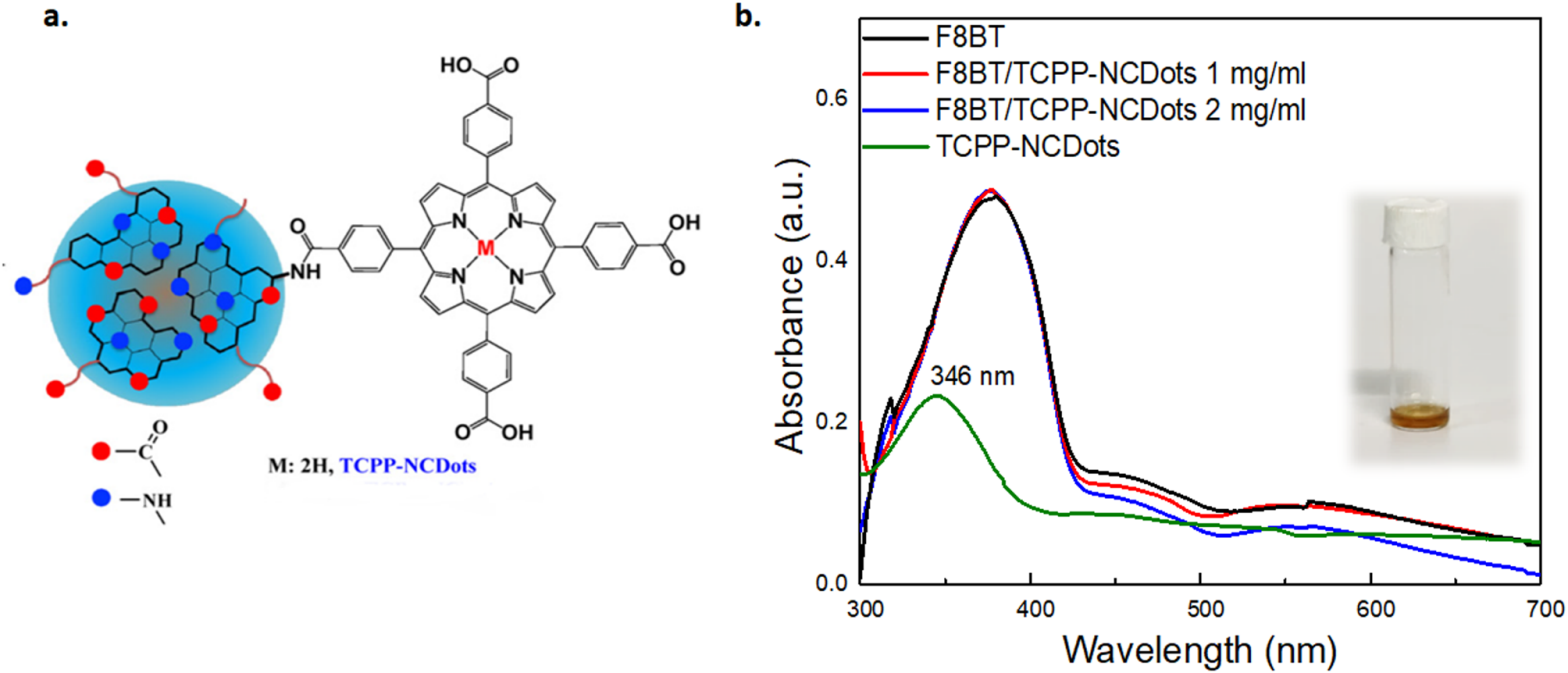
(**a**) Schematic representation of the TCPP-NCDots structure, adapted from Achilleos et al.^62^. (**b**) UV–Vis absorption spectra of a TCPP-NCDots solution in methanol and of F8BT and TCPP-NCDots films at various concentrations.

This study focuses on the charge transfer capabilities of TCPP-NCDots, which are deposited atop the green-yellow emissive co-polymer F8BT. F8BT is selected not only as an established benchmark emitter with well-characterized photophysics and highly reproducible device performance, but also because of its good morphological stability in ambient processing^63^. As shown in Fig. 1a, the covalent linkage of tetra(4-carboxyphenyl)porphyrin (TCPP) with nitrogen-doped carbon dots (NCDots) through amide bond formation creates an electronically conjugated composite nanomaterial. Specifically, the carboxylic acid groups of TCPP react with amino groups on the surface of NCDots and result in the formation of stable –CO–NH- linkage. TCPP exhibits a conjugated π-electron system within its planar tetrapyrrolic macrocycle, consistent with Hückel’s rule for aromaticity, enabling effective π–π* electronic transitions and intermolecular charge delocalization^64–66^. NCDots, composed predominantly of sp^2^-hybridized carbon and contain graphite-like domains capable of supporting π-electron delocalization and charge transport. When covalently linked, the π-systems of both components can interact through orbital overlap, facilitating charge transfer across the interface. The hybrid molecule incorporates a range of bond types including σ-bonds (C–C, C–N, C–H) and π-bonds (C = C, C = O). These interactions synergistically form a nanocomposite that acts as an electronically open channel, potentially enhancing electron injection.

To investigate this synergistic interaction, the optical properties between TCPP-NCDots (ETL) and F8BT (EML) were investigated using UV–Vis absorption spectroscopy, as shown in Fig. 1b. In solution, TCPP-NCDots show a peak at 346 nm. As reported by Ladomenou et al.^61^, this is assigned to the n → π^⁎^ transition of C = O groups in NCDots and appears slightly red-shifted due to TCPP linkage compared to single NCDots. When deposited on F8BT, the absorption spectrum exhibits a minor spectral variation in the main F8BT peak, shifting from approximately 380–378 nm, which however is within the limit od instrumentation error and cannot be considered as a result of F8BT/porphyrin interaction. The secondary peak at 465 nm remains unchanged and is higher than in the hybrid samples. No distinct absorption peaks from TCPP-NCDots are visible in the hybrid film. This suggests the formation of a thin interfacial layer that does not dominate in the optical response^63^. The reduced absorbance of the hybrid samples observed in the 450–600 nm range, may suggest an electronic interaction between the π-conjugated structures of TCPP and F8BT^67^. At their interface, the formation of weak interfacial charge-transfer states can redistribute oscillator strength and lower the effective extinction coefficient of the intrinsic π − π* transitions^68,69^. The resulting interaction appears as a measurable attenuation of the absorption band.

**Fig. 2:**
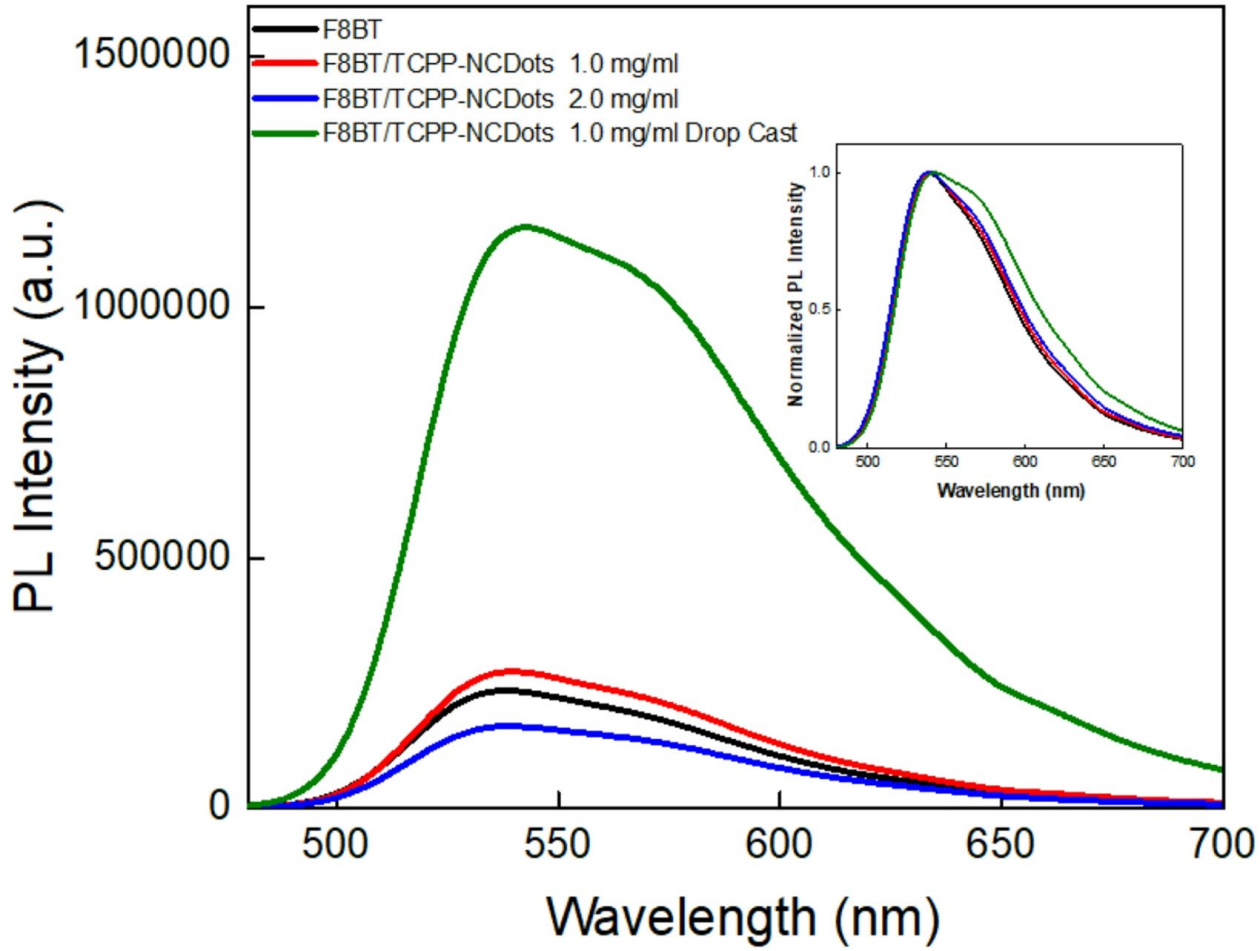
Steady-state photoluminescence (PL) spectra of F8BT and F8BT/TCPP-NCDots films at different concentrations, measured at an excitation wavelength of 380 nm. The inset shows the corresponding normalized PL spectra.

Photoluminescence (PL) measurements were performed to investigate the excited-state interactions in F8BT/TCPP-NCDots films, as presented in Fig. 2. Previous studies^61^, showed that NCDots exhibit excitation-dependent emission, an intrinsic characteristic of carbon dot materials. Achilleos et al.^62^ further reported that TCPP-NCDot conjugates retain this property, while TCPP remains excitation-independent, due to its well-defined electronic states. In Fig. 2, an excitation wavelength of 380 nm was selected to ensure efficient excitation of F8BT. The PL spectra show a maximum emission peak at 540 nm for both pristine F8BT and spin-coated F8BT/TCPP-NCDots films. This indicates that the incorporation of a thin layer of TCPP-NCDots does not significantly alter the fundamental electronic transitions of F8BT. However, the drop-cast sample (meaning that the TCPP-NCDot layer is drpo-casting atop F8BT) exhibits an increase in PL intensity along with a red-shifted additional shoulder. The presence of this shoulder suggests excitonic interactions, potential aggregation effects or energy transfer between F8BT and TCPP-NCDots.

**Fig. 3:**
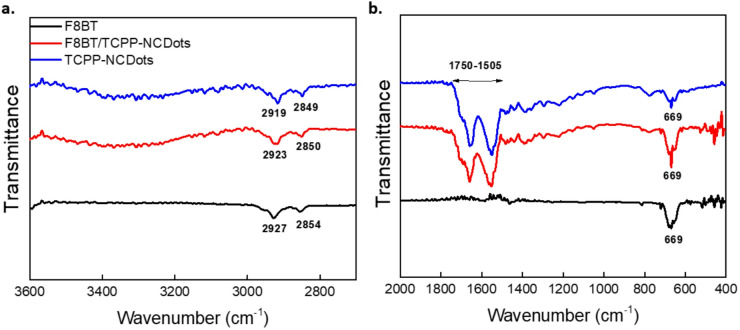
FTIR spectra of pristine TCPP-NCDots, F8BT and F8BT/TCPP-NCDots films in the wavenumber regions (**a**) 3600–2600 cm^−1^ and (**b**) 2000–400 cm^−1^. To enhance spectral comparison, the spectra have been vertically offset along the transmittance axis.

In Fig. 3, the FTIR spectra of F8BT, F8BT/TCPP-NCDots and TCPP-NCDots provide critical insights into their molecular interactions in the wavenumber regions (a) 3600–2600 cm^−1^ and (b) 2000–400 cm^−1^. In the FTIR spectrum of the TCPP-NCDots, a broad absorption band between 3530 and 3000 cm^−1^ corresponds to the stretching vibrations of N–H and O–H groups. This band originates from surface-bound amine and hydroxyl functionalities on the NCDots. The distinct bands observed at 2919 cm^−1^ and 2849 cm^−1^ is attributed to C-H stretching and is consistent with previous reports on CDs synthesized from citric acid^62,70,71^. Additionally, a broad absorption band between 1700 and 1505 cm^−1^ in the TCPP-NCDots spectrum indicates overlapping stretching vibrations and confirms the presence of various functional groups from both TCPP and NCDots. Specifically, the C = O stretching band at 1658 cm^−1^ reflects surface-bound oxygen functionalities^72^. The N–H bending vibration at 1550 cm^−1^ corresponds to amine-related interactions in NCDots, as reported by Achilleos et al^62^. The range 1420–1316 cm^−1^ is consistent with bending vibrations of aliphatic C-H bonds (sp^3^-hybridized) and stretching modes of carboxylate groups^62,70^. The pristine F8BT FTIR spectrum exhibits distinct absorption bands at 2927 cm^−1^ and 2854 cm^−1^, which are assigned to the asymmetric and symmetric stretching vibrations of aliphatic C-H bonds in the octyl side chains^60,73^. A characteristic band observed at 1462 cm^−1^ corresponds to the C = C stretching mode of the aromatic rings and confirms the presence of a π-conjugated system. Additionally, several bands at 814 cm^−1^, 720 and 669 cm^−1^ are assigned to C–H out-of-plane bending vibrations.

In the F8BT/TCPP-NCDots hybrid sample, the FTIR spectrum preserves the sharp and distinct bands in the range 2930–2845 cm^−1^, which are also observed in the pristine F8BT and TCPP-NCDots. These bands appear slightly broadened and shifted. Such spectral changes suggest the presence of intermolecular interactions. In particular, the observed broadening is indicative of hydrogen bonding between F8BT and the functional groups on the surface of TCPP-NCDots^74,75^. The interface also maintains a broad absorption band in the region of 1700–1505 cm^−1^. Within this range, the C = O stretching vibration at 1658 cm^−1^ and the N–H bending mode near 1550 cm^−1^ exhibit mild broadening, supporting further the existence of hydrogen bonding and possibly charge transfer processes^76^. Moreover, the hybrid spectrum retains the C = C stretching vibration at 1462 cm^−1^ and the characteristic C–H rocking modes at 814, 720, and 669 cm^−1^. Also, the 669 cm^−1^ band becomes more intense in the hybrid compared to the pristine spectra. It may indicate further hydrogen bonding and π–π stacking between F8BT and TCPP-NCDots^77^.

**Fig. 4:**
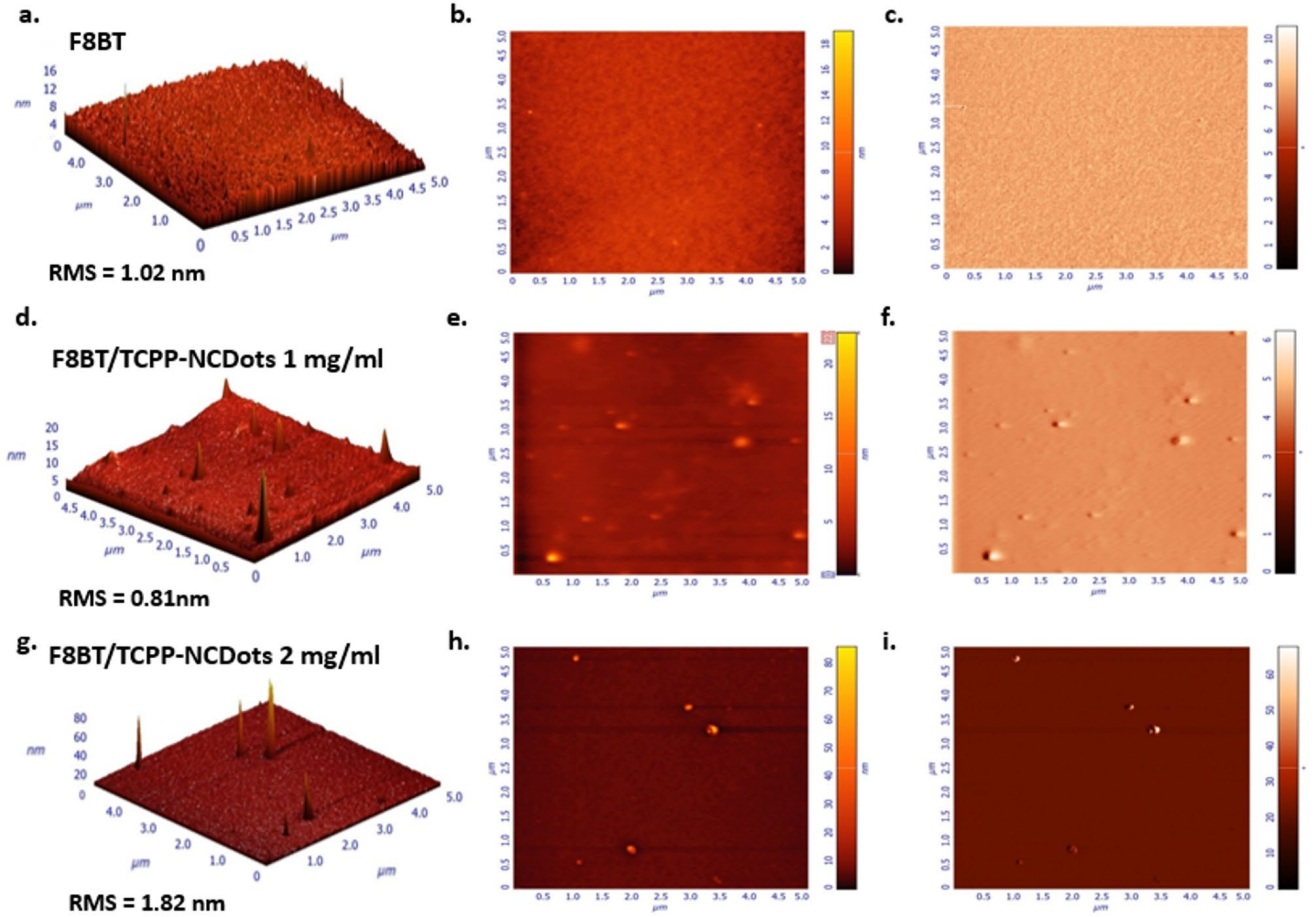
Atomic force microscopy (AFM) surface topographies, including height (3D, left), 2D (middle) and phase (right), of (**a**–**c**) F8BT, (**d**–**f**) F8BT/TCPP-NCDots (1 mg/mL) and (**g**–**i**) F8BT/TCPP-NCDots (2 mg/mL).

Figure 4 presents Atomic Force Microscopy (AFM) images used to analyze the surface morphology and roughness variations of F8BT and F8BT/TCPP-NCDots films. The results confirm that the surface roughness remains consistently low. Figure 4a–c show that the pristine F8BT film exhibits a root mean square (RMS) roughness of 1.02 nm, indicative of a relatively smooth surface. Upon the addition of 1 mg/mL of TCPP-NCDots, as shown in Fig. 4d–f, the roughness decreases to 0.81 nm. Increasing the concentration to 2 mg/mL, as seen in Fig. 4g–i, results in a higher RMS roughness of 1.82 nm. A previous study by our team reported that the incorporation of NCDots generally increases surface roughness, which is consistent with the current findings. Nonetheless, the final film retains a low roughness overall and remains suitable for charge transport mechanisms. This observation supports the decision to avoid concentrations exceeding 2 mg/mL, as higher concentrations would significantly increase roughness, potentially leading to the generation of leakage currents^78^.

**Fig. 5:**
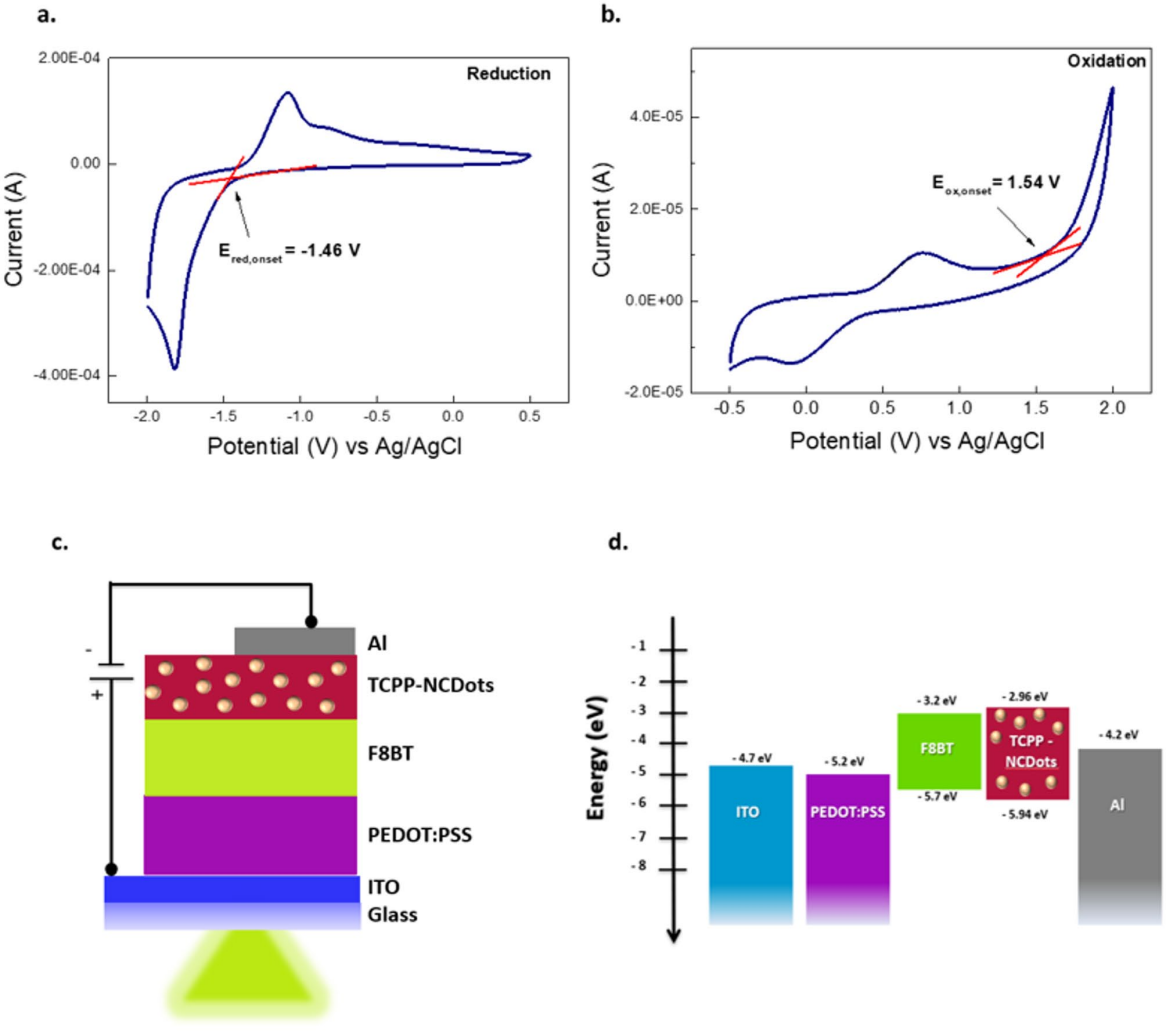
Cyclic voltammetry (CV) measurements were performed in 0.1 M LiClO_4_ dissolved in acetonitrile at room temperature (~ 25 °C). (**a**) CV reduction curve of TCPP-NCDots. (**b**) CV oxidation curve of TCPP-NCDots. (**c**) Schematic representation and (**d**) energy level diagram of the OLED architecture.

To support these, the electrochemical properties of TCPP-NCDots were examined using cyclic voltammetry (CV) in a 0.1 M LiClO_4_/acetonitrile electrolyte. The oxidation and reduction characteristics were systematically analyzed to determine their electronic structure. As shown in Fig. 5a, the reduction process exhibits an onset potential of − 1.46 V, while the oxidation process, presented in Fig. 5b and shows an onset potential of 1.54 V. These values were employed to estimate the highest occupied molecular orbital (HOMO) and lowest unoccupied molecular orbital (LUMO) energy levels using electrochemical empirical equations, as follows:

1 $$ {\mathrm{E}}_{{{\mathrm{HOMO}}}} = - \left( {{\mathrm{E}}_{{{\mathrm{ox}},{\mathrm{onset}}}} + {4}.{4}} \right){\mathrm{eV}}$$

2 $${\mathrm{E}}_{{{\mathrm{LUMO}}}} = - \left( {{\mathrm{E}}_{{{\mathrm{red}},{\mathrm{onset}}}} + {4}.{4}} \right){\mathrm{eV}}$$

The HOMO level of TCPP-NCDots is calculated at − 5.94 eV and a LUMO level at − 2.96 eV, yielding an electrochemical bandgap of 2.98 eV. The slightly higher calculated LUMO level (− 2.96 eV) compared to that of F8BT (− 3.2 eV) supports the efficient electron transfer and the prevention of hole backflow. Figure 5c presents a schematic of the proposed multilayer OLED architecture that incorporates TCPP-NCDots as an electron transport layer (ETL). Indium tin oxide (ITO) serves as the transparent anode that is coated on a glass substrate and enables the hole injection. Poly(3,4-ethylenedioxythiophene) polystyrene sulfonate (PEDOT:PSS) consists the hole transport layer (HTL) and reduces the HOMO energy barrier between ITO and the emissive layer. The emissive layer (EML) is based on F8BT and facilitates charge recombination and electroluminescence. TCPP-NCDots are introduced as the ETL. Aluminum (Al) is finally used as a cathode that enables electron injection into the TCPP-NCDots ETL. The energy level diagram of Fig. 5d depicts the HOMO and LUMO levels of each layer. The incorporation of TCPP-NCDots (HOMO: − 5.94 eV LUMO: − 2.96 eV) bridges the energy gap between F8BT and the Al cathode (− 4.2 eV). With a LUMO level of − 2.96 eV, slightly higher than that of F8BT and a deep HOMO level TCPP-NCDots prevent hole backflow and improve efficient electron transport from the cathode to the EML.

### Impact of TCPP-NCDots on green-yellow OLED performance

**Fig. 6:**
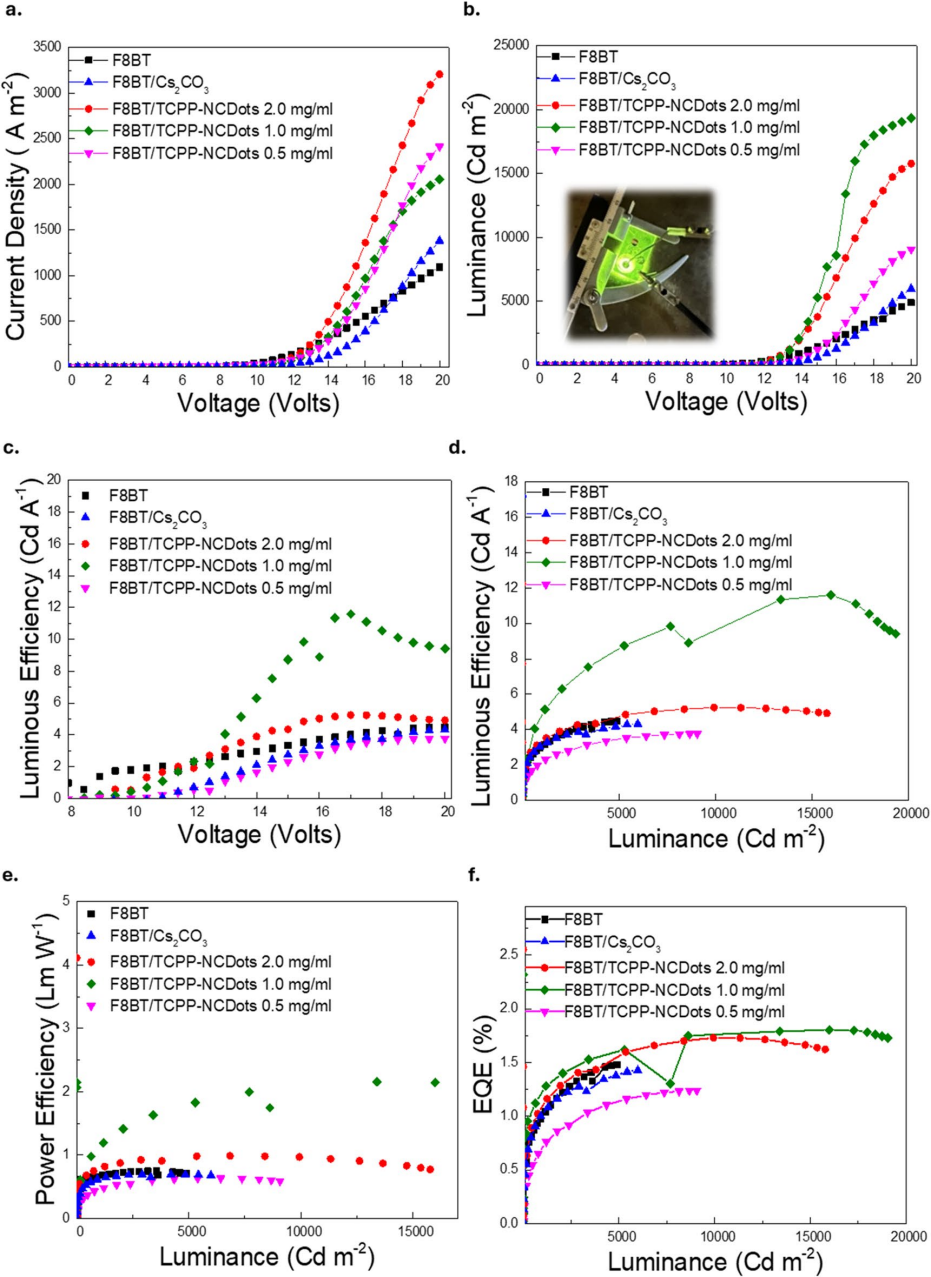
Electrical characterization of OLEDs based on F8BT as EML, incorporating TCPP-NCDots as ETLs at different concentrations. (**a**) Current density vs. Voltage (J-V) characteristics. (**b**) Luminance vs. Voltage (L-V) characteristics, with an inset showing a working OLED device using a TCPP-NCDots ETL at 1 mg/mL concentration. (**c**) Luminous efficiency (LE) vs. Voltage. (**d**) LE and (**e**) Power efficiency (PE) vs. Luminance. (**f**) External quantum efficiency (EQE, %) vs. Luminance.

The electrical properties of OLED devices incorporating TCPP-NCDots as ETLs were evaluated. The current density–voltage (J-V), luminance (L), luminous efficiency (LE), power efficiency (PE) and external quantum efficiency (EQE) measurements are depicted in Fig. 6.The influence of various TCPP-NCDots concentrations on charge injection, transport and recombination efficiency was systematically investigated and was compared to both a pristine F8BT reference device and a device incorporating cesium carbonate, Cs_2_CO_3_ as an alternative ETL.

Figure 6a presents the J-V characteristics of OLEDs with varying TCPP-NCDots concentrations. The reference device exhibits a maximum current density of 1,095 A m^−2^, while the Cs_2_CO_3_-based device increases this value to 1,384 A/m^2^. Incorporation of TCPP-NCDots further enhances current transport, with the 2 mg/mL concentration achieving the highest value of 3,208 A/m^2^. The 1 mg/mL and 0.5 mg/mL devices also exhibit considerable improvements, reaching 3,109 A/m^2^ and 2,415 A/m^2^, respectively. Also, Fig. 6b presents luminance-voltage characteristics for OLEDs with varying TCPP-NCDots concentrations. The 1 mg/mL OLED achieves the highest luminance of 19,344 Cd/m^2^, representing a significant improvement compared to the F8BT device (4,904 Cd/m^2^) and superior to the Cs_2_CO_3_-based device (5,987 Cd/m^2^). The 2 mg/mL device reaches 15,780 Cd/m^2^. The 0.5 mg/mL device, with 9,071 Cd/m^2^, exhibits a moderate improvement over the reference. These variations highlight the role of TCPP-NCDots concentration in optimizing OLED luminance and recombination efficiency.

Figure 6c–d present the luminous efficiency (LE) characteristics at different TCPP-NCDots concentrations, respectively. The optimal performance is again observed for the 1 mg/mL device, which reaches 11.59 Cd/A at 17 V, surpassing the 4.48 Cd/A at 20 V of the pristine F8BT and the 4.32 Cd/A at 20 V of the Cs_2_CO_3_-based device. The 2 mg/mL and 0.5 mg/mL concentrations yield 5.24 Cd/A at 17 V and 3.76 Cd/A at 19 V, respectively. Devices with 1.0 mg/mL and 2.0 mg/mL concentrations in Fig. 6d show suppressed efficiency roll-off, maintaining stable performance even at high luminance. This suggests improved charge balance, reduced exciton quenching and ensures sustained electroluminescence efficiency. Efficiency roll-off is a common challenge in fluorescent OLEDs and occurs due to charge injection imbalances and carrier accumulation, leading to non-radiative recombination losses. The reduced roll-off in TCPP-NCDot-based devices highlights their role in improving overall OLED performance.

Figure 6e highlights the impact of TCPP-NCDots concentration on OLED power efficiency (PE). The 1 mg/mL device achieves the highest PE value of 2.16 lm/W and then, the 2 mg/mL OLED follows with 0.99 lm/W. Lower concentrations result in reduced power efficiency, with the 0.5 mg/mL device reaching 0.63 lm/W. In contrast, the reference F8BT device achieves 0.74 lm/W, while the Cs_2_CO_3_-based OLED shows a slightly lower value of 0.69 lm/W, both remaining well below the TCPP-NCDot-optimized configuration. In parallel, Fig. 6f presents the external quantum efficiency (EQE_max_) as a function of luminance for each TCPP-NCDots concentration. The 1 mg/mL and 2 mg/mL devices exhibit the highest values of 1.80% and 1.73% , respectively, surpassing the reference F8BT OLED (1.48%) and the Cs_2_CO_3_-based device (1.43%). The 0.5 mg/mL device drops to 1.24%, respectively. The electroluminescence (EL) in Figure S1 shows that TCPP-NCDots do not introduce significant spectral shifts across different TCPP-NCDots concentrations and their incorporation suggests minimal impact on the emission wavelength.

The integration of TCPP-NCDots as ETLs demonstrates a profound impact on OLED performance, with the 1 mg/mL device exhibiting the most pronounced enhancements. Compared to the pristine F8BT-based device, luminance experiences a remarkable 294% increase. Luminous efficiency rises by around 159%, while power efficiency is enhanced by about 192%. Moreover, the EQE_max_ increases by nearly 22% percent, attaining 1.80%, confirming that TCPP-NCDots facilitate more effective photon generation and extraction. A small, abrupt drop in the EQE curve of the 1.0 mg/mL device at high brightness originates from a temporary optical collection instability in the measurement setup and specifically in fiber coupling, which caused a local deviation in the recorded luminance. This feature is therefore considered a measurement artifact and not related to the intrinsic performance of the device.

### Performance degradation of F8BT/ TCPP-NCDots in OLEDs

To assess the long-term stability of the OLED devices, the samples were stored under ambient conditions in an open environment for four days. After this period, electrical measurements repeated to evaluate any changes in charge transport, luminance and efficiency and are presented in Fig. S2.

The reference OLED device exhibits a notable decline in performance after being exposed to environmental conditions for four days. The maximum current density value decreases by 9%, from 1,095 to 996 A/m^2^. A 86% drop in luminance is observed, with its maximum value reaches from 4,904 Cd/m^2^ to 681 Cd/m^2^. Both the maximum LE and PE values decrease by 85%. Also, it presents the most severe decline in EQE that drops by 84% from 1.48 to 0.23%. The Cs_2_CO_3_-based device also shows substantial degradation. Maximum current density decreases by 23%, from 1,384 to 1,066 A/m^2^. Luminance suffers a dramatic 98% reduction, reaching only 130 Cd/m^2^. Efficiency degradation is extreme, with LE and PE decreasing by 97%, reaching 0.12 Cd/A and 0.02 lm/W. EQE falls by 97%, from 1.43 percent to 0.04%, confirming very weak stability of Cs_2_CO_3_ as an ETL in this system.

The 2 mg/mL OLED device also experiences performance degradation but maintains a higher stability compared to the references. The maximum current density reduces by 16%, from 3,208 to 2,710 A/m^2^. The maximum luminance value drops by 73%, from 15,780 Cd/m^2^ to 4,250 Cd/m^2^. Both the maximum LE and PE values decrease by 70%. Also, it is characterized by a 69% decline, from 1.73 to 0.53% and suggests exciton quenching over time.

The 1 mg/mL OLED demonstrates the highest stability and best performance retention. Maximum current density decreases by 11%, from 2,056 to 1,830 A/m^2^. Luminance reduces by 64%, from 19,344 to 6,943 Cd/m^2^, yet remains the highest among all devices after four days. LE decreases by 67%, from 11.59 to 3.83 Cd/A, and PE reduces by 72%, from 2.16 to 0.60 lm/W, while still maintaining superior performance to other devices. EQE declines by 30%, from 1.80 to 1.26%, confirming significantly improved charge injection and recombination stability compared to the references, F8BT-based and Cs_2_CO_3_-based devices.

The 0.5 mg/mL OLED device shows moderate degradation but remains more efficient than the reference. Maximum current density lowers by 4%, from 2,415 to 2,505 A/m^2^. The maximum LE falls by 31%, from 9,071 to 6,274 Cd/m^2^. The maximum LE and PE values correspond to a 34% and 35% reduction, respectively. Meanwhile, the 0.5 mg/mL device depicts the slowest EQE degradation, with a 33% decrease from 1.24 to 0.83%. This efficiency decline suggests a more controlled degradation process, as it remains superior to higher-concentration devices and indicates improved charge balance over time. After four days of environmental exposure, OLEDs incorporating TCPP-NCDots retain superior EL intensity compared to the reference, as depicted in Fig. S3.

All TCPP-NCDots-based OLEDs exhibit superior performance compared to the reference device. They achieve enhanced initial efficiency and prolonged operational stability. The concentration of TCPP-NCDots dictates both parameters with notable impact. At higher concentrations, such as 2 mg/mL, charge injection and exciton formation are markedly improved, yielding elevated EQE in the initial phase. However, the efficiency decline over four days suggests intensified non-radiative recombination. Conversely, in lower concentrations, such as 0.5 mg/mL, the attenuation of degradation rates indicate superior morphological integrity and mitigated exciton quenching. However, the lower initial EQE values imply that electron transport may be less efficient, potentially due to limited charge injection. The 1 mg/mL device represents the optimal balance and provides high initial efficiency (1.80%). Also, it maintains a moderate degradation rate in EQE_max_ (30%) over four days. This suggests that at this concentration, charge transport, radiative exciton recombination and operational stability are well-balanced, minimizing efficiency roll-off while maintaining optoelectronic properties.

## Discussion

This study introduces TCPP-functionalized nitrogen-doped carbon dots (TCPP-NCDots) as a high-performance electron transport layer (ETL) in OLEDs that effectively enhances electron transport and improved device performance. UV–Vis spectroscopy confirms the successful functionalization of TCPP-NCDots, while PL analysis demonstrates that the emissive properties of F8BT are preserved upon their integration. The FTIR spectra further validate the good and compatible interfacial coupling between the emissive layer and the ETL. AFM analysis unveils that the overall film morphology remains suitable for efficient charge transport with optimized general roughness that may contribute to reduced leakage currents. Electrochemical analysis shows that TCPP-NCDots possess energetically aligned HOMO (− 5.94 eV) and LUMO (− 2.96 eV) levels between the F8BT and Al interface. OLED devices incorporating TCPP-NCDots exhibit significantly enhanced current density, luminance, luminous efficiency (LE), and external quantum efficiency (EQE) relative to reference devices. At an optimal concentration of 1 mg/mL, the ETL yields a well-balanced charge injection profile, achieves the highest EQE and significantly minimizes the roll-off phenomenon. Long-term operational stability studies demonstrate that the devices retain superior performance after four days of ambient exposure. By combining porphyrins and nitrogen-doped carbon dots into a single hybrid structure for ETL, this study elucidates their synergistic role in optimizing the charge transport properties. These findings offer critical insights into the development of solution-processed, environmentally sustainable electron transport materials for next-generation optoelectronic applications.

## Methods

### Fabrication process of OLED devices

The fabrication of OLED devices commenced with glass substrates coated with indium-tin oxide (ITO with a sheet resistance of 15–25 Ω/sq. These ITO-coated substrates were procured from Sigma-Aldrich, Athens, Greece. Prior to device fabrication, the substrates underwent a sequential ultrasonic cleaning process in deionized water, acetone, and isopropyl alcohol (IPA), each for 10 min, to eliminate organic and particulate contaminants. Following the cleaning procedure, the substrates were dried using nitrogen (N_2_) gas and subjected to UV-Ozone treatment for 20 min, enhancing surface wettability and ensuring optimal adhesion for subsequent layers. A 1.3 wt% dispersion of PEDOT:PSS (poly(3,4-ethylenedioxythiophene)-poly(styrenesulfonate)) in water, sourced from Sigma-Aldrich, was utilized as the hole transport layer (HTL). The solution was first filtered through a polyvinylidene fluoride (PVDF) filter with a 0.45 mm pore size to remove aggregates. The filtered PEDOT:PSS solution was subsequently spin-coated onto the ITO substrates at 6000 rpm for 40 s. The coated substrates were then annealed on a hotplate at 110 °C for 45 min, facilitating solvent evaporation, improving film morphology, and enhancing conductivity for efficient charge injection. The green-yellow copolymer F8BT (poly[(9,9-dioctylfluorenyl-2,7-diyl)-alt-co-(1,4-benzo-{2,10,3}-thiadiazole)]), obtained from American Dye Source (ADS 233 YE), Quebec, Canada, was used as the emissive layer. A 10 mg/mL chloroform-based solution of F8BT was spin-coated on the top of PEDOT:PSS layer at 1200 rpm for 40 s. The coated substrates were then annealed at 85 °C for 10 min. Following emissive layer deposition, a TCPP-NCDots solution in methanol was spin-coated on top of F8BT at 2000 rpm for 40 s. This layer was applied without an additional annealing step. A Cs_2_CO_3_ interlayer was prepared by spin-coating a 2-methoxyethanol-based solution of Cs_2_CO_3_ onto the F8BT layer at 2000 rpm for 40 s. The film was deposited without any subsequent annealing treatment and was used as a second reference device. To complete the OLED fabrication, a 150 nm aluminum (Al) cathode electrode was deposited via thermal evaporation. Each OLED device featured an active surface area of 12.56 mm^2^ in order to provide a consistent platform for electrical characterization.

### Characterization techniques

UV–Vis absorption spectra were obtained using a Perkin Elmer Lambda 40 UV–Vis spectrometer (Vamvakas-Scientific Equipment, Athens, Greece). Steady-state photoluminescence (PL) measurements were conducted using a **Shimadzu RF-6000 Spectrofluorophotometer**. Fourier-transform infrared (FTIR) spectroscopy was performed using a Bruker Tensor 27 spectrometer (Interactive, Athens, Greece), with a DTGS detector. The surface morphology of the films was examined through atomic force microscopy (AFM) in tapping mode, utilizing an NT-MDT AFM system from LaborScience SA (Athens, Greece). Cyclic voltammetry (CV) measurements were performed using a VersaSTAT 4 potentiostat (Megalab SA, Athens, Greece) with a three-electrode system, consisting of an indium tin oxide (ITO) working electrode, a platinum wire counter electrode and an Ag/AgCl reference electrode. The electrolyte solution used was 0.1 M LiClO_4_ in acetonitrile. For electrical characterization, current density–voltage-luminance (J–V–L) measurements were conducted using a Keithley 2400 Source-Meter (Vector Technologies, Athens, Greece) in voltage mode with fixed incremental steps. Electroluminescence (EL) spectra were recorded with a calibrated BPW34 Si PIN photodiode (Vector Technologies, Athens, Greece).

## Supplementary Information

Below is the link to the electronic supplementary material.

Supplementary Material 1

## Data Availability

The data are available by M.V. per reasonable request.
