# Supplementary material for: Porphyrin-nitrogen carbon dot composites for high-performance organic light-emitting diodes
Source: Sci Rep. 2026 Jan 29;16:5507. doi: 10.1038/s41598-026-35190-5 (PMC12886781; doi:10.1038/s41598-026-35190-5)
Supplement: Supplementary file 1 — Supplementary Material 1 [file 41598_2026_35190_MOESM1_ESM.docx]

**Porphyrin-Nitrogen Carbon Dot Composites for High-Performance Organic Light-Emitting Diodes**

Zoi Georgiopoulou^1,2^, Maria Eleni Rizou^1^, Apostolis Verykios^1^, Anastasia Soultati^1^, Georgios Chatzigiannakis^1,2^, Theodoros M. Triantis^1^, Alexander Chroneos^3,4,*^, Kalliopi Ladomenou^5^, Athanassios G. Coutsolelos^6, 7*^, Maria Vasilopoulou^1,*^

^1^Institute of Nanoscience and Nanotechnology, National Center for Scientific Research ‘Demokritos’, Agia Paraskevi 15310, Athens,Greece.

^2^ Solid State Physics Section, Department of Physics, National and Kapodistrian University of Athens, Panepistimioupolis, Zografos 15784, Athens, Greece.

^3^Department of Materials, Imperial College, London SW7 2AZ, United Kingdom

^4^Department of Electrical and Computer Engineering, University of Thessaly, 38221 Volos, Greece

^5^Hephaestus Laboratory, School of Chemistry, Faculty of Sciences, Democritus University of Thrace, GR-65404 Kavala, Greece.

^6^Laboratory of Bioinorganic Chemistry, Department of Chemistry, University of Crete, Voutes Campus, 70013, Heraklion, Crete, Greece

^7^Foundation for Research and Technology (FORTH), Institute of Electronic Structure and Laser (IESL), 70013, Heraklion, Greece

[^*^acoutsol@uoc.gr](mailto:*acoutsol@uoc.gr) (Athanassios G. Coutsolelos)

[^*^m.vasilopoulou@inn.demokritos.gr](mailto:*m.vasilopoulou@inn.demokritos.gr) (Maria Vasilopoulou)

[*alexander.chroneos@imperial.ac.uk](mailto:*alexander.chroneos@imperial.ac.uk) (Alexander Chroneos)

**Supplementary Material**

**Fig.~~ure~~ S1.** Electroluminescence (EL) spectra of **OLED devices incorporating ETLs based on TCPP-NCDots** at different concentrations (**2.0 mg/ml, 1.5 mg/ml, 1.0 mg/ml, and 0.5 mg/ml**), measured at **11 V**, respectively.


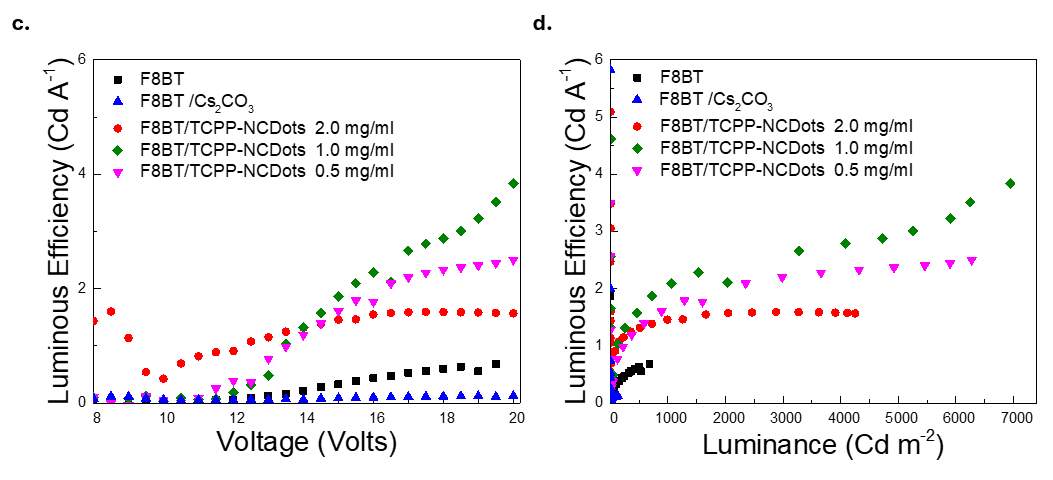

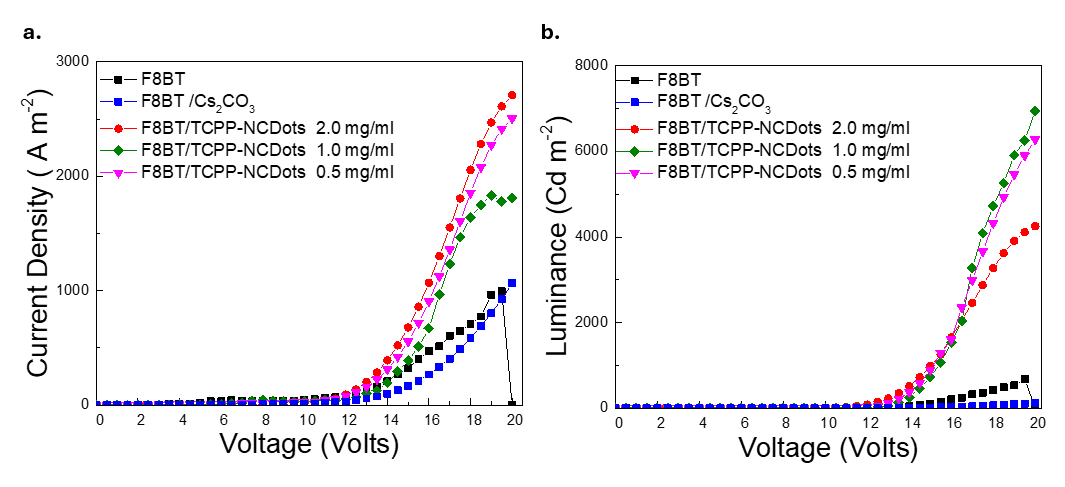

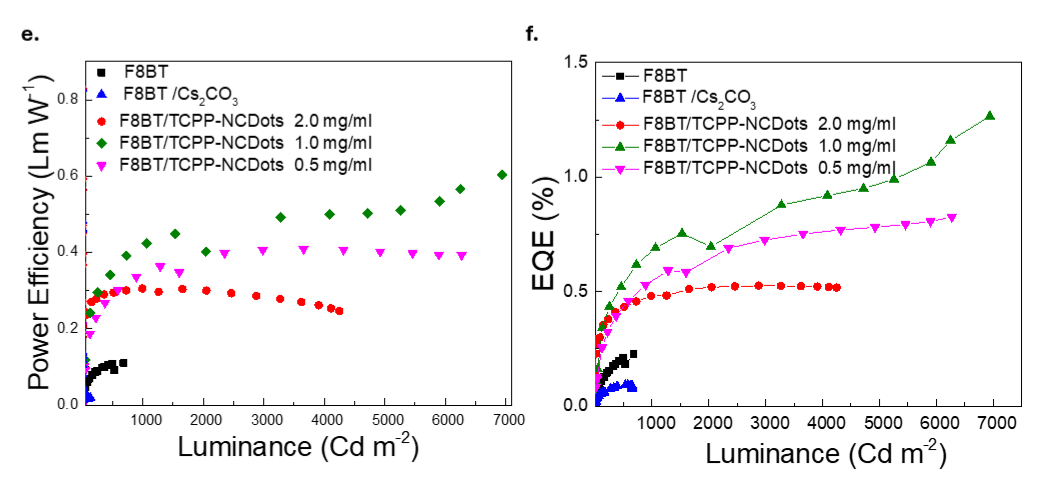


**Fig.~~ure~~ S2. Electrical characterization of OLEDs based on F8BT as the EML, incorporating TCPP-NCDots as ETLs at various concentrations, measured after four days in a free-space environment.** (a) Current density versus Voltage (V). (b) Luminance versus Voltage. (c) Luminous efficiency (LE) versus Voltage. (d) LE versus Luminance and (e) Power efficiency (PE) versus Luminance. (f) External Quantum Efficiency (EQE, %) versus Luminance. The F8BT and F8BT/Cs₂CO₃ devices were used as references for comparison.


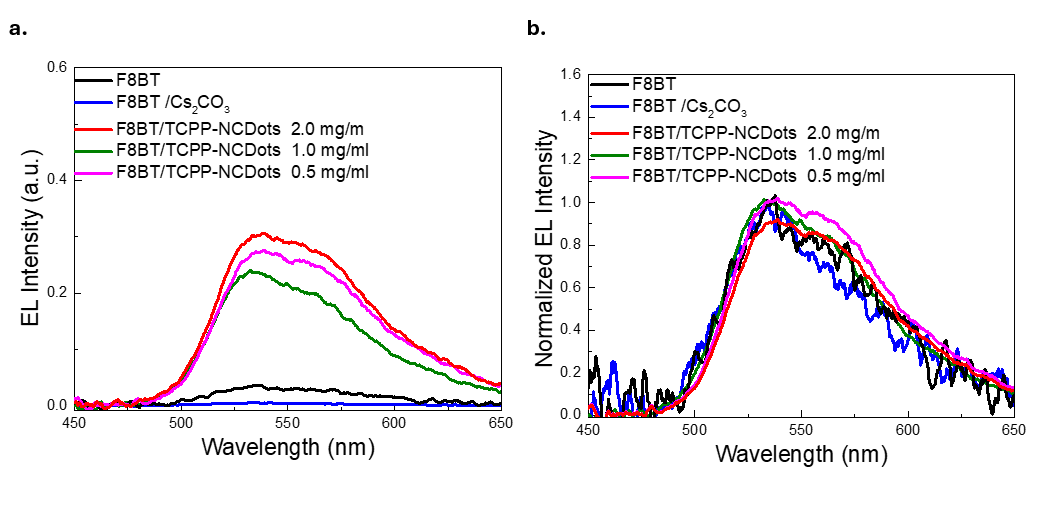


**Fig.~~ure~~ S3.** (a) Electroluminescence (EL) and (b) Normalized EL spectra of OLED devices with ETLs based on TCPP-NCDots at different concentrations (2.0 mg/ml, 1.0 mg/ml, and 0.5 mg/ml), recorded at 11 V and measured after four days in a free-space environment. The F8BT and F8BT/Cs₂CO₃ devices were used as references for comparison.
